# Contrast-induced nephropathy in ITU patients: outcomes of a university hospital re-audit

**DOI:** 10.1186/cc9519

**Published:** 2011-03-11

**Authors:** K Lam, T Chan, R Lowsby, J Walker

**Affiliations:** 1 Royal Liverpool University Hospital, Liverpool, UK

## Introduction

Contrast-induced nephropathy (CIN) is a significant and preventable cause of renal failure associated with increased mortality, hospital stay and long-term haemodialysis. Critically ill patients have increased risks of developing CIN due to pre-existing disease and sepsis. A university hospital audit in 2007 found that 22.2% of ITU patients had significant rises in creatinine following intravenous contrast medium (IVCM). In 2008, IVCM guidelines were implemented trust-wide to detect patients with pre-existing renal impairment and provide guidance for pre-optimisation and prophylactic measures depending on CKD stage, including early renal team involvement. A re-audit assessed the impact of IVCM guidelines in decreasing the incidence of CIN in ITU.

## Methods

ITU patients who received IVCM for CT studies from March to December 2010 were identified. Patients on haemodialysis pre-contrast or who died within 48 hours post-contrast administration were excluded. Pre-contrast (within 48 hours) and post-contrast (48 to 72 hours) creatinine levels were analysed. CIN was defined as an increase in serum creatinine exceeding 25% or 44 μmol/l from baseline within 3 days of administration of contrast media in the absence of alternative causes.

## Results

Ninety patients were identified. Ten patients who required haemodialysis pre-contrast or died within 48 hours post-contrast were excluded. Mean age was 59 years (range 25 to 89 years) with a male:female ratio of 46:34. Fourteen (17.5%) patients had significant rises in creatinine post-contrast. Patients who died within 48 hours had ruptured AAA, severe sepsis, ischaemic bowel, and so forth.

## Conclusions

The incidence of CIN has decreased to 17.5% in medical and surgical ITU patients since the introduction of the IVCM guidelines.

